# Super-Stable Metal–Organic Framework (MOF)/Luciferase Paper-Sensing Platform for Rapid ATP Detection

**DOI:** 10.3390/bios13040451

**Published:** 2023-04-01

**Authors:** Héctor Martínez-Pérez-Cejuela, Maria Maddalena Calabretta, Valerio Bocci, Marcello D’Elia, Elisa Michelini

**Affiliations:** 1 Department of Chemistry “Giacomo Ciamician”, Alma Mater Studiorum-University of Bologna, Via Selmi 2, 40126 Bologna, Italy; 2 Department of Analytical Chemistry, University of Valencia, C/Dr. Moliner, 50, 46100 Burjassot, Spain; 3 Center for Applied Biomedical Research (CRBA), Azienda Ospedaliero-Universitaria Policlinico S. Orsola-Malpighi, 40138 Bologna, Italy; 4 Istituto Nazionale di Fisica Nucleare (INFN) Sezione di Roma, 00185 Rome, Italy; 5 Scientific Police Centre for Emilia-Romagna Region, 40123 Bologna, Italy; 6 Health Sciences and Technologies Interdepartmental Center for Industrial Research (HSTICIR), University of Bologna, 40126 Bologna, Italy

**Keywords:** ATP, luciferase, biosensor, metal–organic frameworks, bioluminescence, paper-sensing

## Abstract

Adenosine triphosphate (ATP) determination has been used for many decades to assess microbial contamination for hygiene monitoring in different locations and workplace environments. Highly sophisticated methods have been reported, yet commercially available kits rely on a luciferase–luciferin system and require storage and shipping at controlled temperatures (+4 or −20 °C). The applicability of these systems is limited by the need for a secure cold chain, which is not always applicable, especially in remote areas or low-resource settings. In this scenario, easy-to-handle and portable sensors would be highly valuable. Prompted by this need, we developed a bioluminescence paper biosensor for ATP monitoring in which a new luciferase mutant was combined with a metal–organic framework (MOF); i.e., zeolitic imidazolate framework-8 (ZIF-8). A paper biosensor was developed, ZIF-8@Luc paper sensor, and interfaced with different portable light detectors, including a silicon photomultiplier (SiPM) and smartphones. The use of ZIF-8 not only provided a five-fold increase in the bioluminescence signal, but also significantly improved the stability of the sensor, both at +4 and +28 °C. The ATP content in complex biological matrices was analyzed with the ZIF-8@Luc paper sensor, enabling detection down to 7 × 10^−12^ moles of ATP and 8 × 10^−13^ moles in bacterial lysates and urine samples, respectively. The ZIF-8@Luc sensor could, therefore, be applied in many fields in which ATP monitoring is required such as the control of microbial contamination.

## 1. Introduction

Adenosine triphosphate (ATP), the energy-carrying molecule present in all living cells, can be considered to be a signature of the presence of biological matter; therefore, it is highly useful for hygiene monitoring on surfaces or in water [1].

The majority of methods for ATP detection rely on the use of firefly luciferase (FLuc), an ATP-dependent enzyme, which catalyzes the oxidation of D-luciferin (D-LH_2_) in the presence of ATP, Mg(II) and molecular oxygen, leading to the emission of light [2]. As the emission of light is proportional to the ATP content and, consequently, to the number of cells, several bioluminescence (BL) methods have been developed, allowing microbial contamination and the presence of biological contaminants such as blood and skin cells to be assessed within a few minutes [3,4]. Highly sensitive portable light detectors have been developed based on photomultiplier tubes (PMTs), photodiodes, complementary metal oxide semiconductors (CMOSs) and silicon photomultipliers (SiPMs) [5]. The detection of ATP also has important clinical implications; intra-cellular ATP levels are correlated with several neurological diseases and microbial ATP in urine is a marker of urinary tract infections [6,7,8]. Alternative solutions have been reported to improve the sensitivity and stability of reagents, including microfluidic chips interfaced with an SiPM detector, enabling detection down to 8 nM of ATP [9]. Smartphone-based paper sensors relying on a lyophilized “nano-lantern” provided a limit of detection (LOD) of 10^−14^ mol [10]. This system was applied to the analysis of urinary microbial ATP [6]. In addition to the biosensor analytical performance, other important aspects such as the cost-effectiveness and the sustainability (i.e., the use of green reagents and materials, in agreement with the principles of green analytical chemistry [11], or better, with more holistic white analytical chemistry [12]) should also be considered.

The issue of stabilizing the luciferase enzyme at non-controlled temperatures has not been solved. Marketed ATP detection kits must be stored at +4 °C or −20 °C. To address this challenge, significant efforts have been focused on the generation of new luciferase mutants with improved thermal and pH stability [13].

As an alternative strategy, luciferase can be immobilized onto nanosized supports; i.e., magnetic nanoparticles [14], graphite [15], silica [16] and other nanomaterials [17,18]. Metal–organic frameworks (MOFs) have also been recently reported for this purpose [19,20]. MOFs were shown to be highly suitable for hosting biomolecules [21,22,23]. Their structure is based on a porous network composed of metal ions and organic ligands [24]. The selection of adequate synthesis precursors has a huge impact on the chemical surface, biocompatibility and stability [25]. The flexibility and versatility of their use has prompted their application in different fields, including biosensing, bioimaging and drug-delivery systems [26,27,28]. Very recently, the possibility of developing paper biosensors combining enzymes and MOFs has also been explored with colorimetric detection. Kou et al. reported a MOF nanoreactor paper for the real-time colorimetric detection of glucose and uric acid [29]. Thanks to its intrinsic nature, facile synthesis and high stability, zeolitic imidazolate framework-8 (ZIF-8) is one the most studied MOFs [30,31]. ZIF-8 is constructed from Zn(II) ions and 2-methylimidazole ligands, synthesized under mild conditions in short periods of time. Despite the popularity of this material and its benefits, the combination of ZIF-8 with luciferase has only recently been explored [32]. We demonstrated that the immobilization of luciferase onto ZIF-8 not only favored enzyme protection towards denaturing conditions (e.g., the presence of organic solvents, an acidic pH and repeated freeze/thawing processes), but also increased the storage stability at room temperature. Although other MOF supports (e.g., UiO-66 and MIL-101) could be used to host biomolecules, the intrinsic properties of ZIF-8 make it the most suitable candidate for biosensor development [32].

Here, we report a BL paper-based device for ATP detection in which, for the first time, a firefly luciferase mutant, BgLuc, was immobilized onto ZIF-8 and integrated into a paper-sensing platform. The selection of a stable luciferase mutant and ZIF-8 enabled us to address the typical issues that hamper the applications of biosensors to real-life needs; i.e., a lack of stability and sensitivity as well as cost-effectiveness. This approach could represent a realistic alternative to commercial kits for ATP quantification, with the additional advantages of full portability, a low cost and low volumes of reagents/samples. In addition, an accessible and simple photodetector relying on SiPM [33] was selected to provide an all-in-one device. To demonstrate the applicability of this biosensor to the analysis of real biological samples, we analyzed the ATP content of urine samples, detecting down to 8 × 10^−13^ moles. The method was also applied for the detection of ATP from bacterial lysate (*E. coli*), showing the effectiveness of the MOF to improve the analytical performance of the biosensor, which outperformed the same biosensor configuration with the free luciferase.

## 2. Materials and Methods

### 2.1. Reagents and Materials

Zinc nitrate hexahydrate, 2-methylimidazole (HMIM), Trizma^®^ hydrochloride, magnesium chloride hexahydrate BioXtra, ATP (disodium salt hydrate), tryptone, sucrose, bovine serum albumin, *E. coli*-competent cells (JM109) and all other reagents were obtained from Sigma-Aldrich (Milwaukee, WI, USA). Methanol (MeOH) (99.9% *v/v*) was purchased from VWR International Eurolab (Milan, Italy). Beetle luciferin potassium salt was obtained from Promega (Madison, WI, USA). BgLuc recombinant protein was produced as previously described [13]. Whatman^®^ number 1 CHR cellulose chromatographic paper (W1) was obtained from GE Healthcare (Chicago, IL, USA). The office printer ColorQube 8400 (Xerox, Norwalk, CT, USA) was used for the wax printing. For the 3D-printed holders, black acrylonitrile butadiene styrene (FormFutura, Nijmegen, The Netherlands) and polylactic acid (Anycubic, Shenzhen, China) resins were used. All media and materials used in these processes were autoclaved for 20 min at 120 °C. MilliQ (MQ) water was used during all the work and it was obtained from a Rephile Bioscience Millipore purification system (reference A+). All other reagents used were of an analytical grade.

### 2.2. Portable Light Detectors

For the BL measurements, two portable light detectors were used; a portable SiPM device (LuminoSiPM) [34] and a smartphone (OnePLus A6013; OnePlus, Shenzen, China). The LuminoSiPM was an all-in-one system embedding a Hamamatsu MPPC 13360-1325CS SiPM sensor containing 2668 GM-APD cells (pixel pitch of 25 µm) as sensitive matrix with a photosensitive available area of 1.3 × 1.3 mm and 25% maximum photon detection efficiency at 450 nm. The OnePlus smartphone as equipped with a 16 MP Sony Exmor RS IMX 519, BSI CMOS 1/2.6″ color sensor with 1.22 μm pixels and an ƒ/1.7 aperture and a secondary sensor; a 20 MP Sony Exmor RS IMX 376K, BSI CMOS 1/2.8″ color sensor with 1.0 μm pixels and an ƒ/1.7 aperture. A Replicator 2X 3D printer (Makerbot, Boston, MA, USA) was used for fabricating the mobile holder (Figure S1).

### 2.3. Synthesis of the ZIF-8@Luc Biocomposite

The synthesis of the biocomposite was based on a previously reported procedure [32,35], with minor modifications. Briefly, ZIF-8 was synthesized by adding a solution of 50 mL of MeOH containing 19.76 mmol of HMIM dropwise into 50 mL of a MeOH solution containing 2.47 mmol of Zn(NO_3_)_2_×6H_2_O. A white dispersion was formed and the dispersion was incubated under stirring for 24 h in atmospheric conditions. The resulting solid was separated by centrifugation at 15,000× *g* for 15 min and rinsed twice with 40 mL MeOH before drying at 80 °C for 12 h. The solid material was ground and sieved to collect the fraction between 100 and 200 µm to improve the homogeneity.

Next, 0.25 mg of homogeneous ZIF-8 powder was mixed in a 90 µL volume of 50 mM Tris-HCl (pH 7.8). The mixture was sonicated in an ice-water bath for 15 min, then incubated for 60 s. A 10 µL volume of a 3.0 mg mL^−1^ luciferase solution was added and incubated for 30 min at 4 ± 1 °C under orbital shaking (200 rpm). Next, the ZIF-8@Luc was separated by centrifuging at 15,000× *g* for 5 min and the pellet was washed twice with 50 µL of 50 mM Tris-HCl (pH 7.8). A 100 µL volume of a 50 mM Tris-HCl (pH 7.8) solution was added and the biocomposite was homogenized by vortexing for 60 s and an ultrasonic bath for 120 s. The BL measurements were carried out in a 96-well microplate as follows: a 5 μL volume of 10 mM MgCl_2_ was mixed with 5 μL of 2 mM ATP, and a 6 μL volume of 0.1 mg mL^−1^ ZIF-8@Luc dispersion was added. Next, 6 μL of 1 mM D-LH_2_ was automatically dispensed and the emission spectra were recorded for 25 min with a 500 ms integration time.

### 2.4. Fabrication of the ZIF-8@Luc Paper Sensor

Once the synthesis and characterization of the biocomposite were carried out, the paper-based biosensor was designed and fabricated. W1 paper was used as a host platform to immobilize the ZIF-8@Luc biocomposite. Circular areas with a diameter of 5 mm were created by depositing wax ink (hydrophobic part) on the paper. After heating at 100 °C for 2 min, the wells were individually cut and sealed with adhesive tape to prevent solvent leakage. After the assembly of the paper support, the lyophilization was conducted using an Alpha 1-2 LD plus freeze-dryer (Martin Christ, Osterode am Harz, Germany) to obtain the ready-to-use paper biosensor. R18 cryoprotectant was selected to perform the freeze-drying process [36]. The optimized freeze-drying process was performed for 2 h at −50 °C with the homogenized mixture comprising 5:1 (*v*/*v*) of 0.25 mg L^−1^ ZIF-8@Luc:R18 cryoprotectant (6 µL total volume) for each paper sensor. After this process, the individual sensing papers were kept at −20 °C before further use.

### 2.5. BL Signal Acquisition and Data Treatment

*Benchtop luminometer.* The BL measurements were performed with a benchtop Varioskan LUX (Thermo Scientific, Waltham, MA, USA) equipped with an automatic injection. The BL measurements of the biocomposite in a liquid format were performed in white 384-well plates (Greiner Bio One North America, Monroe, NC, USA) with 0.5 s acquisitions. The BL measurements of the ZIF-8@Luc immobilized on paper were performed by cutting the single paper wells and transferring them onto the wells of a white 96-well plate. The BL signals were acquired for 20 min after a D-LH_2_ automatic injection, and the spectra were acquired at the maximum emission intensity (2 min after the D-LH_2_ injection) from 350 to 750 nm with a 0.5 nm bandwidth. All the measurements were performed in triplicate and repeated at least two times. In a common BL analysis, a 6 µL volume of 1.0 mM D-LH_2_ was added to a mixture of 6 µL of 0.25 mg mL^−1^ ZIF-8@Luc dispersion, 5 µL of 10 mM MgCl_2_ and 5 µL of 2 mM ATP before the BL measurement.

*BL measurements with LuminoSiPM.* Specific Microsoft Windows software was used to acquire signals with ArduSiPM [34] (latest stable version v. 1.0). All the measurements were performed in triplicate and the signal was acquired for 15 min, unless otherwise stated. A total of 900 numerical acquisitions were recorded, with data sampling cycles of 1 s, in the form of counts per second (cps) for any sample/replicate. The resulting data were obtained in a CSV format.

*BL measurements with the OnePlus smartphone.* For the smartphone acquisition, the paper-based sensors containing lyophilized ZIF-8@Luc were analyzed using the secondary sensor (20 MP camera) of the OnePlus 6 smartphone with 30 s integration and ISO 3200. The image analysis was performed with ImageJ free software (National Institutes of Health, Bethesda, MD) to quantify the signal intensity over the region of interest (ROI) (100 × 100 pixels). The signals were expressed as relative light units (RLUs).

*ZIF-8@Luc paper assay format.* In a typical BL analysis, 4 µL of 10 mM Mg(II) and 4 µL of 1 mM D-LH_2_ were added to a previously lyophilized ZIF-8@Luc on paper (6 µL of 5:1 mixture *v*/*v*, 0.25 mg mL^−1^ ZIF-8@Luc:R18). A 2 µL volume of the sample (or ATP standard solution) was added and the signal measurements were performed, as described before according to a different detector. GraphPad Prism v.8 software (GraphPad Software, La Jolla, CA, USA) was used to analyze and plot the data.

### 2.6. Characterization of the ZIF-8@Luc Paper-Sensing Platform

*Chemical and structural characterization.* ZIF-8 was characterized to check the correct formation of the 3D network. Scanning electron microscopy (SEM) and transmission electron microscopy (TEM) images were obtained with a SEM microscope, model Hitachi S-4800 (Ibaraki, Japan), and a TEM microscope, model JEM-1010 JEOL, coupled to an AMT RX80 digital camera (Akishima, Japan), respectively. Nitrogen isotherms (adsorption/desorption) were acquired using an S5 Micromeritics ASAP2020 instrument (Norcross, GA, USA) at 77 K. The Brunauer–Emmet–Teller (BET) theory was applied to estimate the active surface area, taking into account the low-pressure range. The powder X-ray diffraction (p-XRD) spectrum was acquired with a D8 Advance A25 diffractometer (Bruker, Hamburg, Germany). Attenuated total reflection Fourier-transform infrared (FT-IR) spectra were obtained with a Bruker FT-IR spectrometer (Bremen, Germany), model Tensor 27, coupled to a nine-reflection diamond/ZnSe DuraDisk plate.

*Bioluminescence characterization.* Several parameters of the final sensing platform were optimized to achieve the best analytical performance and highest storage stability at different temperatures (−20, +4 and +28 °C). Dose–response curves for ATP were obtained using both portable light detectors, the smartphone-integrated CMOS and the LuminoSiPM device. LODs were calculated as the blank signal plus three times the standard deviation.

The Michaelis–Menten constants (Km) for D-LH_2_ and ATP were determined using saturating levels of D-LH_2_ (0.001–5 mM) and Mg-ATP (0.0002–20 mM). The obtained Km values were the average of three different experiments (±standard deviation, SD).

The precision of the method was assessed by calculating the relative standard deviation (RSD) between the different biosensor responses obtained in different conditions; for instance, the intra-batch precision was assessed using the same batch of sensing platforms (n = 6) during the same day and conditions. The inter-batch precision was assessed using different batches of MOF synthesis of purified luciferase and paper sensors fabricated on different days (n = 6).

### 2.7. Real Sample Analysis and Stability Studies

We assessed the suitability of the method to detect ATP in biological samples by analyzing bacterial lysates. *E. coli* cultures were grown at 37 °C for 16 h (approx. OD 600 = 0.9) with orbital stirring at 200 rpm in an LB medium. A total of 1 mL of cell culture was then centrifuged (10 min at 13,000 rpm), resuspended in 100 µL of a 1:1 *v*/*v* LB medium:lysis buffer and incubated for 10 min under orbital shaking (60 rpm at 25 °C). The ATP analyses were performed according to Section 2.5. The assay was performed using both the ZIF-8@Luc paper-sensing platform and free luciferase.

Urine samples were selected to assess the feasibility of the sensing platform to analyze complex clinical samples. First, morning urine samples were collected from fasting healthy volunteers in sealed containers and refrigerated at 4 °C for later use. Recovery studies were performed on four urine samples previously centrifuged at 18,000 rpm for 5 min spiked with low and medium ATP concentrations (0.02 and 2.00 mM, respectively).

For the stability studies, three syntheses were performed at day 0 and the resulting sensing platforms were kept in darkness at different temperatures (−20, +4 and +28 °C). The BL signal was measured on different days according to the previous section.

## 3. Results and Discussion

**Figure 1:**
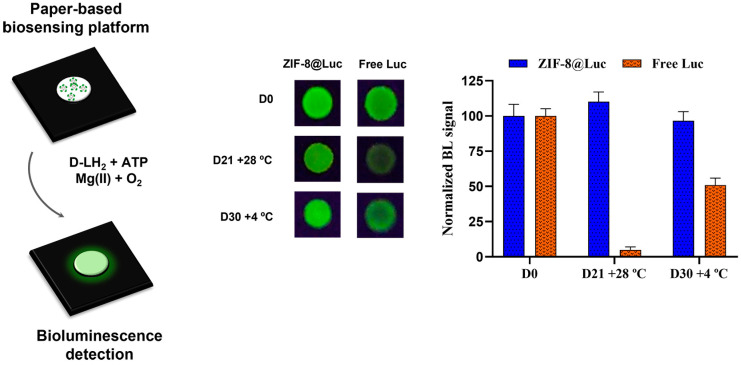
ZIF-8@Luc paper-sensing platform for adenosine triphosphate (ATP) quantification. Representative images of the ZIF-8@Luc paper acquired with the smartphone and stability studies of ZIF-8@Luc in comparison with the Luc paper sensor without ZIF-8.

We recently reported the advantageous combination of ZIF-8 and luciferase for improving the analytical performance and stability of luciferase-based assays [32]. Encouraged by these results, we explored the applicability of these nanobiocomposites for the development of ready-to-use BL paper biosensing platforms (Figure 1).

### 3.1. ZIF-8@Luc Paper Sensor Fabrication

To develop a paper-based biosensor exploiting the combination of ZIF-8 and luciferase, different strategies were explored, including the simple drop-casting technique, followed by the evaporation of the solvent and freeze-drying of the ZIF-8@Luc biocomposite with D-luciferin. In both cases, a significant loss of BL signal was reported, with a 60% decrease in the BL signal in the latter case (Figure S2). Therefore, lyophilization on paper of the bare ZIF-8@Luc biocomposite was selected (Figure S3), optimizing the amount of R18 cryoprotectant and freeze-drying time (from 2 to 8 h) (Figure S4) [10,36]. R18, by saturating the biocomposite surface via the non-specific adsorption of sugars and proteins, prevents damage to the enzyme caused by ice crystals [37]. Different R18 cryoprotectant concentrations between 0 and 30% (*v*/*v*) were evaluated (Figure S4). Although the MOF had a protective behavior (Figure S3) towards freeze/thawing processes [32], R18 (1:5 *v*/*v* ratio) enhanced the luciferase stability, leading to a significant signal improvement (4.5-fold higher than the control without R18). Higher concentrations (~30% *v*/*v*) led to a BL signal decrease (around 20%), most likely due to the reduction in available active sites in the luciferase. Therefore, the addition of R18 as a cryoprotectant was further used at the ratio 5:1 *v*/*v*. Concerning the freeze-drying times, no significant differences in the BL signal were observed in the tested conditions (Figure S4), but a lower variability of the measurements was observed with 2 h freeze-drying (7.5% vs. ca. 20% obtained with longer freeze-drying processes). The use of 5:1 (*v*/*v*) of the 0.3 mg L^−1^ ZIF-8@Luc:R18 medium and a freeze-drying process of 2 h were selected. It is worth mentioning that the binding forces between ZIF-8 and free luciferase were mainly based on hydrogen bonding and hydrophobic interactions, including both van der Waals forces and Π-stacking [32].

### 3.2. Characterization of the ZIF-8@Luc Paper Sensor

**Figure 2:**
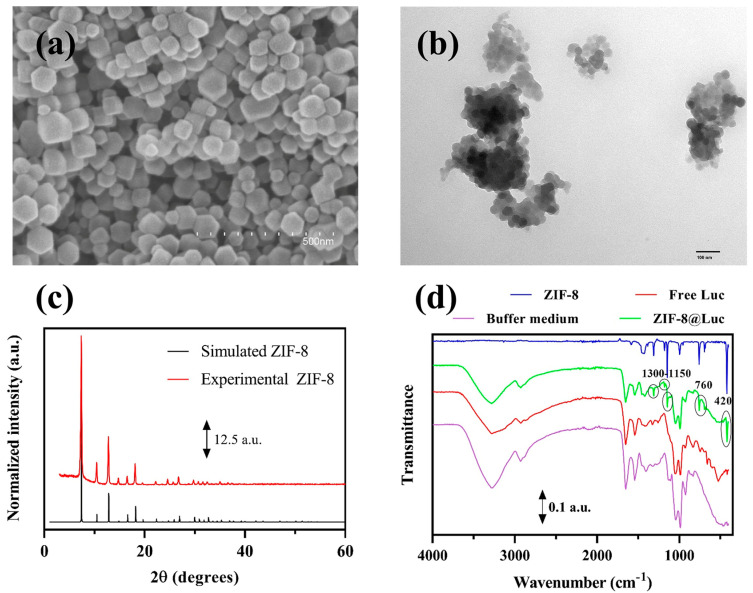
Chemical and structural characterization studies: (**a**) Scanning electron microscopy (SEM) image; (**b**) Transmission electron microscopy (TEM) image; (**c**) Powder X−ray diffraction (PXRD); (**d**) Fourier-transform infrared spectroscopy (FT−IR) spectra; details are available in Materials and Methods section.

*Chemical and structural characterization.* SEM and TEM images were acquired to check the correct synthesis of both ZIF-8 and the biocomposite, showing the typical octahedral shape (Figure 2) [32,35]. Furthermore, the nitrogen isotherms from the ZIF-8 support were obtained (Figure S5). It exhibited typical Type I isotherms, confirming the microporous nature of this MOF. The active surface area, which was calculated by using the Brunauer–Emmett–Teller (BET) equation, was ~1400 ± 25 m^2^g^−1^, which was in agreement with previous studies [35]. Next, the ZIF-8 XRD spectrum was acquired and compared with the simulated one. All the characteristic diffraction peaks matched between the spectra, confirming the crystallinity of this material. FT-IR spectra were collected from the pristine material and after the sensor manufacture (Figure 2d). The ZIF-8@Luc paper showed peaks around 1300–1150, 760 and 420 cm^−1^, which could be identified in the ZIF-8 spectrum, indicating its presence. These findings not only indicated the presence of both materials after the freeze-drying process, but also the integrity of the composite at room temperature. Taking into account these results, the nanobiocomposite sensor was further investigated in terms of the BL response.

**Figure 3:**
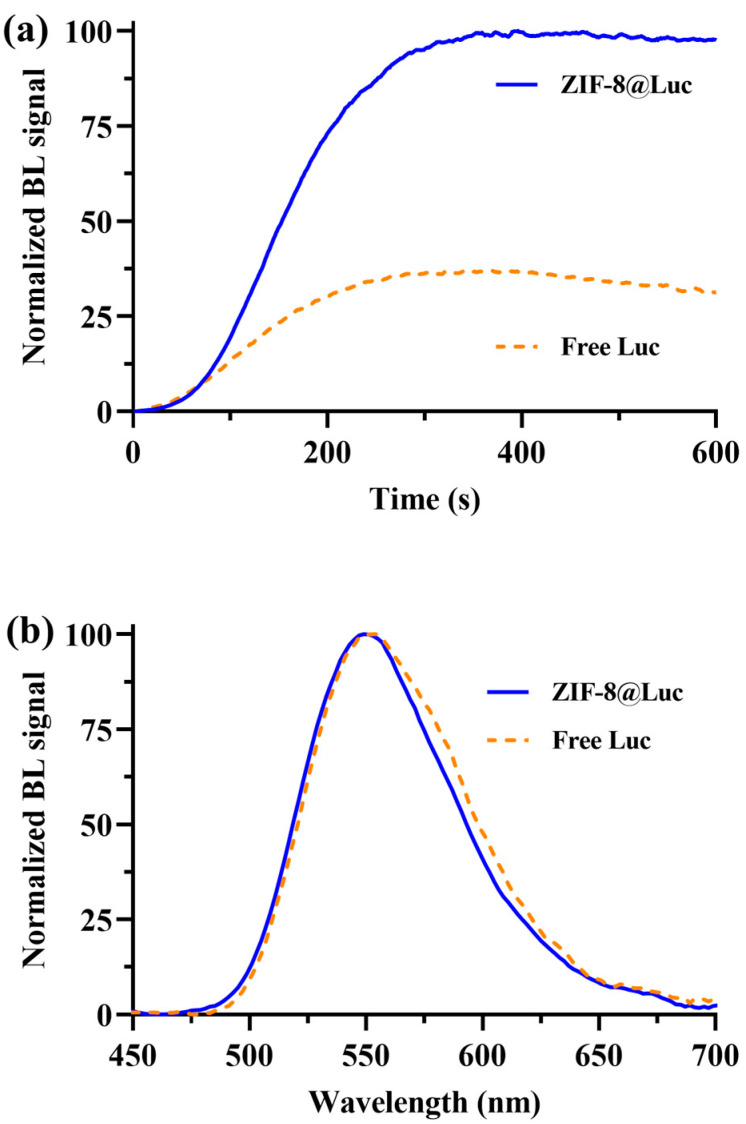
Characterization of the bioluminescence (BL) emission properties of ZIF-8@Luc paper sensor in comparison with the free luciferase mutant obtained with a benchtop luminometer with acquisition 10 min after D-LH_2_ automatic injection: (**a**) BL kinetics; (**b**) BL emission spectra obtained with ZIF-8@Luc sensor and free luciferase mutant in the same conditions.

*Bioluminescence characterization*. BL emission kinetics and the emission spectrum of the ZIF-8@Luc sensor were measured (Figure 3). The immobilization of luciferase onto ZIF-8 provided a signal enhancement in the first 300 s of about 65% in the tested conditions (Figure 3a). This could be caused by the self-assembly of the conformation of ZIF-8@Luc, which favors signal enhancement in glow-type kinetics [38,39]. The ZIF-8@Luc emission spectra were compared with those of the free luciferase mutant, showing emission maxima at 549 nm and 553 nm with a half bandwidth of 74 and 76 nm, respectively (Figure 3b), demonstrating that the attachment to the ZIF-8 network had no influence on the BL luciferase emission behavior.

**Figure 4:**
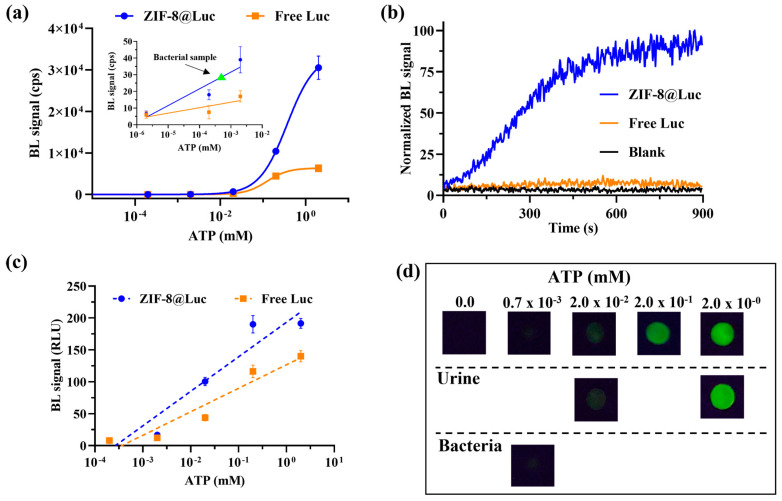
(**a**) Dose–response curve for ATP acquired by LuminoSiPM. The inset represents the linear correlation in the lower range of the BL signal vs. log (ATP conc.) and the points represent the BL average from 3 replicates; (**b**) BL kinetics with LuminoSiPM detection ATP conc. 1 µM; (**c**) dose–response curves for ATP obtained with the smartphone; (**d**) representative images of ZIF-8@Luc paper sensor response in the presence of different ATP concentrations and real samples obtained with the smartphone. The average of four independent replicates is represented in both graphs. The green triangle corresponds with the concentration of the analyzed bacterial lysate sample. A few error bars (Figure 4a,c) are not visible because they are smaller than the symbol size.

Dose–response curves for ATP were obtained using the optimized procedure described in the Materials and Methods section and two different portable light detectors, the smartphone-integrated CMOS and a LuminoSiPM device. These two detectors were chosen with the goal of having either an instrument-free paper sensor that did not require additional instrumentation, except for the smartphone of the end user, or a cost-effective all-in-one device. The latter option, although requiring an additional instrument for the photon detection, had a non-negligible advantage; i.e., it did not depend on the smartphone-integrated camera, which may have affected the reproducibility of the results and the performance of the sensor. In fact, the LuminoSiPM integrated an SiPM sensor connected to an Arduino Due board, with the possibility of transferring data in real-time to a PC via a USB port. Figure 4 shows the dose–response curves for ATP obtained with the two detectors. The obtained LODs for ATP were 8 × 10^−13^ moles (0.4 µM) and ~4 × 10^−12^ moles (2 µM) for ZIF-8@Luc and Free Luc, respectively.

Intra- and inter-batch precisions were evaluated by analyzing two concentrations of ATP (0.02 mM and 2.0 mM) by measuring the BL signal at different timepoints (300, 600 and 900 s). Paper sensors fabricated using the same batch of purified luciferase, MOF synthesis and reagents were used for the intra-batch precision, and paper sensors obtained on different days with different batches of purified luciferase and reagents were used for the inter-batch precision. In all cases, the RSD obtained was between 8 and 20% (Table 1).

The Km for ATP and D-LH_2_ for the ZIF-8@Luc paper sensor was also evaluated (Table 2). Both Kms for ATP and D-LH_2_ obtained with the ZIF-8@Luc paper were lower compared with the Kms obtained with the free luciferase immobilized on paper, suggesting an improved accessibility for the substrate. This could be explained by the large surface area of ZIF-8, which could host the luciferase and provide a better enzyme conformation and improved accessibility.

Interestingly, the presence of ZIF-8 decreased the LODs for ATP to 400 nM using both portable detectors (Table 2), most likely due to a lower Km and the protective action of ZIF-8 on the enzyme. As all measures were conducted at room temperature (25 °C), a higher dark noise was reported for the LuminoSiPM; this could be improved by cooling the sensor with a Peltier chamber down to −10 °C.

### 3.3. ATP Quantification in Real Samples

To further confirm the applicability of the ZIF-8@Luc paper sensor, real samples were analyzed using bacterial cultures and spiked urine samples. Bacterial lysate from *E. coli* was first chosen as a model of microbial contamination. As shown in Figure 4a,c, only the ZIF-8@Luc paper sensor provided an adequate analytical performance for analyzing the samples with concentrations of ATP as low as 0.7 × 10^−3^ mM (7×10^−12^ moles) (corresponding with OD 600 = 0.9). The LOD obtained with the free luciferase paper sensor was not adequate.

Recovery studies were also performed by spiking urine samples with known amounts of ATP (2 × 10^−2^ mM and 2.00 mM). The results, expressed as an average from 4 different samples (Table 3), confirmed the suitability of the sensor to analyze urine samples. We hypothesized that ZIF-8 could also exert a “clean-up function” and retain a few potential interfering compounds present in the biological matrix (Figure 4b,d). This aspect deserves further investigations because the possibility of analyzing complex biological samples without any treatment is of vital importance for producing biosensors suitable for real-life needs.

### 3.4. Stability Studies

**Figure 5:**
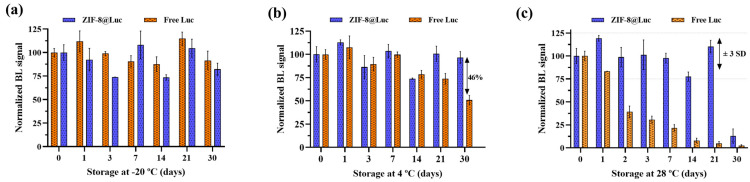
Stability studies of the ZIF-8@Luc paper sensor after storage at −20 °C (**a**), +4 °C (**b**) and +28 °C (**c**).

Regarding stability, we assessed the possibility of storing the sensing paper in different conditions (−20 °C, +4 °C and +28 °C). It is well-known that storage at −20 °C is not a feasible solution in several cases and drastically reduces the number of potential applications (e.g., shipping the biosensor to remote low-resource settings). The combination of ZIF-8 with luciferase improved the stability in all three tested temperatures (Figure 5 and Figure S6). Surprisingly, in presence of ZIF-8, storage up to 21 days at room temperature (28 °C) did not lead to a significant BL decrease; meanwhile, the free luciferase had lost about 95% of the initial BL signal. Furthermore, after 30 days of storage at +4 °C, the ZIF-8@Luc paper sensor and the paper sensor with free luciferase showed 97% and 51% of the remaining BL signal, respectively. Stability was assessed up to 2 months at −20 and +4 °C (Figure S6). Regarding the +4 °C storage study, the sensor based on ZIF-8@Luc presented 96% and 90% of the BL remaining activity compared with 51% and 43% obtained by the free luciferase after 1 and 2 months, respectively. This evidence was also observed, albeit to a lesser extent, in the study performed at −20 °C, where the ZIF-8@Luc sensor retained a BL activity of 99% after 2 months of storage, but only 58% of the BL remaining activity was observed in the case of free luciferase.

The ZIF-8@Luc paper sensor was compared with recently published biosensors in terms of the analytical performance, cost-effectiveness and sustainability [9,10,40,41,42]. For instance, Wang and collaborators [40] reported a method based on glassy carbon electrodes with a LOD around 0.08 µM and an acquisition time of 10 min, which were both comparable with our sensor. However, our sensor was fully portable and it did not require either washing steps or deproteinization steps for the sample treatment. Zhang et al. reported an interesting paper sensor based on the hydrolysis of S-methyl-L-cysteine, generating an odor with slightly higher LODs (1.6–10 µM) [41]. The authors stated that their first approach (sealed-tube sample preparation protocol) was too complex for in-field use, which could be an important drawback. Another ATP paper sensor achieved a lower LOD (3.8×10^−14^ moles); however, as with other sensors [9,42] based on luciferin–luciferase systems, they used commercial kits as reagents for the sensor fabrication. This made the sensors much more expensive than our solution, which could easily be reproduced in any laboratory without the need for special equipment. In addition, the ZIF-8@Luc paper sensor showed a significant stability at room temperature, which, together with the portability of the final device and its cost-effectiveness (<0.20 EUR per sensor), made this approach a feasible and green alternative for ATP sensing in field analyses.

## 4. Conclusions

We developed a portable paper-based ATP biosensor, which was coupled to portable detectors, a LuminoSiPM and smartphone-integrated CMOS. To the best of our knowledge, this is the first bioluminescent paper sensor combining ZIF-8 and luciferase. The sensor showed appealing features in terms of the analytical performance, with high stability and activity, cost-effectiveness, a light weight, and sustainability. The Michaelis–Menten constants for ATP and D-LH_2_ were estimated and the presence of MOFs indicated that the accessibility towards the substrate was enhanced, probably due to the stabilization of a preferred conformation. Interestingly, the LOD for ATP obtained with the ZIF-8@Luc paper sensor was five times lower than that obtained with the Luc paper sensor without ZIF-8. The analysis of real samples using *E. coli* lysates and recovery studies performed in urine samples showed an adequate analytical performance for real-life applications. An excellent stability was reported at room temperature, with an almost unaltered response after 21 days. This finding agreed with a recent report in which a fusion protein between tamavidin2 (an avidin analog) and Gaussia luciferase were stabilized by ZIF-8 and could be stored at room temperature for up to 6 months [43]. The present method, therefore, represents a new straightforward tool for the analysis of real samples, requiring only 2 µL of sample and 30 min for the complete analysis. The ZIF-8@Luc paper sensor could be applied to many different fields in which ATP monitoring is required such as the control of microbial contamination [44]. Our results serve as guide for future works in the biosensing field, taking advantage of the use of MOFs and bioluminescent enzymes on paper supports.

## Figures and Tables

**Table 1:** Precision studies for paper biosensors obtained with different acquisition times (300, 600 and 1200 s).

ATP Conc. (mM)	Time (s)	Precision (RSD, %)	
		Intra-Batch (n = 6) ^1^	Inter-Batch (n = 6) ^2^
0.02	300	11.0	9.8
	600	12.4	8.0
	900	16.7	15.2
2.00	300	18.5	9.7
	600	19.9	11.2
	900	19.5	8.2

^1^ Intra-batch values (n = 6, on the same day) using the same Luc purification, MOF synthesis and reagent batches. ^2^ Inter-batch values (n = 6, on different days) using different Luc purification, MOF synthesis and reagent batches.

**Table 2:** Michaelis–Menten constants and limits of detection (LOD) obtained with ZIF-8@Luc paper sensor and free luciferase. Km was determined with a benchtop luminometer.

	Km (µM)		LOD (nM)	
	ATP	D-LH_2_	LuminoSiPM	Smartphone
ZIF-8@Luc	834 ± 50	240 ± 50	400 ± 20	389 ± 40
Free Luc	2774 ± 300	301 ± 25	1995 ± 300	666 ± 45

**Table 3:** Determination of adenosine triphosphate (ATP) in spiked urine samples.

Urine	Added (mM)	Found (mM)	Recovery (%)
Male	0.02	0.018 ± 0.005	93.5
	2.00	1.83 ± 0.17	91.8
Female	0.02	0.019 ± 0.002	99.5
	2.00	1.72 ± 0.14	86.0

## Data Availability

The data presented in this study are available on request from the corresponding author.

## Supplementary Materials

The following supporting information can be downloaded at: https://www.mdpi.com/article/10.3390/bios13040451/s1, Figure S1: The 3D-printing devices (holders and adaptors) for (A) LuminoSiPM detector and (B) smartphone camera; Figure S2: Optimization of the freeze-drying. Figure S3: Kinetic study performed with the LuminoSiPM detector and the paper sensor without R18 cryoprotectant. Figure S4: Method optimization: freeze-drying process. Figure S5: Nitrogen adsorption/desorption isotherms of ZIF-8. Figure S6: Long-term stability studies of ZIF-8@Luc and free luciferase-based biosensors [45].
