# Supplementary material for: Super-Stable Metal–Organic Framework (MOF)/Luciferase Paper-Sensing Platform for Rapid ATP Detection
Source: Biosensors (Basel). 2023 Apr 1;13(4):451. doi: 10.3390/bios13040451 (PMC10136344; doi:10.3390/bios13040451)
Supplement: Supplementary file 1 [file biosensors-13-00451-s001.zip › biosensors-2273811-supplementary.pdf]

biosensors

Supplementary Material

Super-Stable Metal–Organic Framework (MOF)/Luciferase Paper-Sensing Platform for Rapid ATP Detection

Héctor Martínez-Pérez-Cejuela [1,2], Maria Maddalena Calabretta [1,3], Valerio Bocci [4], Marcello D'Elia [5] and Elisa Michelini [1,3,6,*]

1. Experimental Section

1.1. Fabrication of 3D Printed Supports for the Detection Platforms

Regarding LuminoSiPM detector case, all the components were designed with FreeCAD software. A black box was fabricated with polylactic acid (PLA) polymer to protect the sample from external light during the data acquisition. Additionally, a sample-holder (squared shape, 17 × 12 mm) was designed with UV 405 nm sensitive resin in order to accommodate the sample in front of the detector at 2 mm away from the sensitive surface. To increase the reproducibility, reference points in both dark box and sample holder were performed with complementary holes and pins, respectively (Figure S1A).

A mobile-adaptor and sample holder were also fabricated to increase the feasibility and reproducibility in the image acquisition (Figure S1B) following the protocols previously optimized with slight modifications [45]. Briefly, a SketchUp Free browser-based 3D design platform (Trimble, CA, USA) was used to build the 3D structures and the resulting files were saved in stereolithography file format (.STL). Next, the 3D models were sliced in thin layers with the software MakerWare v.2.4 (Markerbot) and the new file was saved in .X3G format. In this case, black acrylonitrile-butadiene-styrene (ABS) copolymer was used at 250 μm layer resolution (10 % infill). The entire mobile adaptor was designed for hosting the Oneplus A6013 smartphone with an ergonomic and robust structure (75 × 75 mm, 80 mm height and 100 g total weight). For this purpose, 2 different cases were constructed, which were connected by four small neodymium magnets (N52, 6 × 2 mm of diameter and thickness, respectively). This holder fits perfectly with the mobile-adaptor slot and avoids external light contamination. The distance between the smartphone camera and the sensing platform was kept constant at 5 cm allowing the correct focusing of the image acquisition.

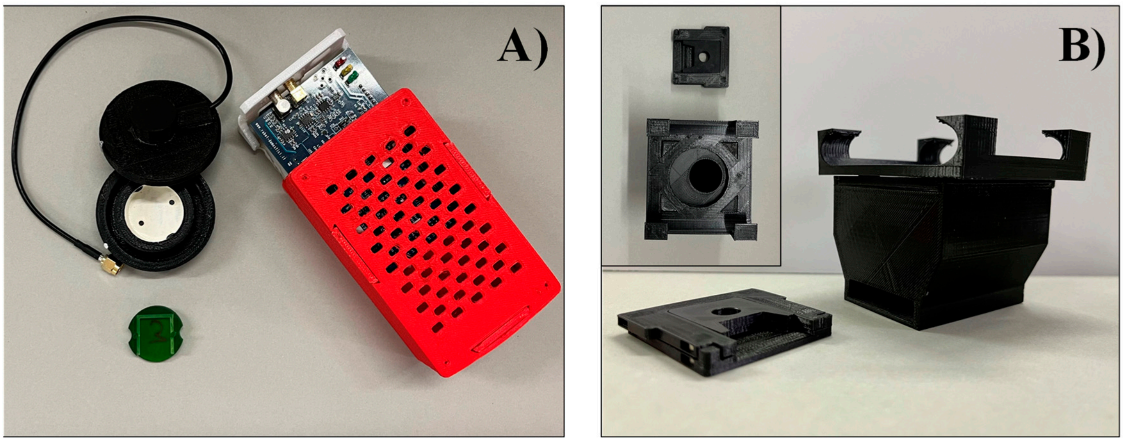

Figure S1. 3D-printing devices (holders and adaptors) for A) LuminoSIPM detector and B) smartphone camera.

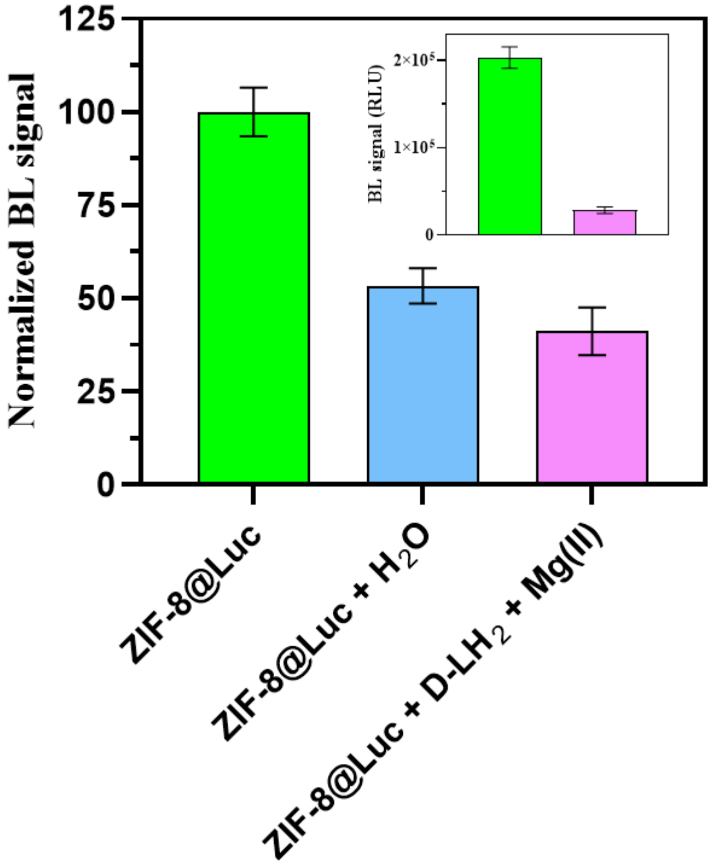

Figure S2. Optimization of the freeze drying optimization. Total volume evaporated: Green bar 6 μL (5 μL of 0.3 mg L^{-1} ZIF-8@Luc and 1 μL of R18 cryoprotectant); Blue bar 14 μL (5 μL of 0.3 mg L^{-1} ZIF-8@Luc, 1 μL of R18 cryoprotectant and 8 μL H_2O); and Purple bar 14 μL (5 μL of ZIF-8@Luc, 1 μL of R18 cryoprotectant and 5 μL 1 mM D-LH_2 and 3 μL of 10 mM Mg(II)). Inset represents the evaporation of the same volume with and without all the reagents needed for the BL reaction (changing the redispersion buffer).

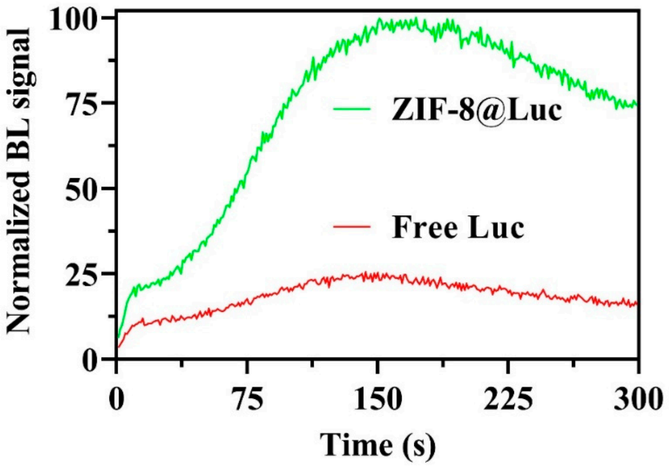

Figure S3. Kinetic study performed with the LuminoSIPM detector and the paper sensor without R18 cryoprotectant ($V_{evaporated}$: 6 μL, 4 h at -50 ºC). More details can be found in Materials and Methods Section.

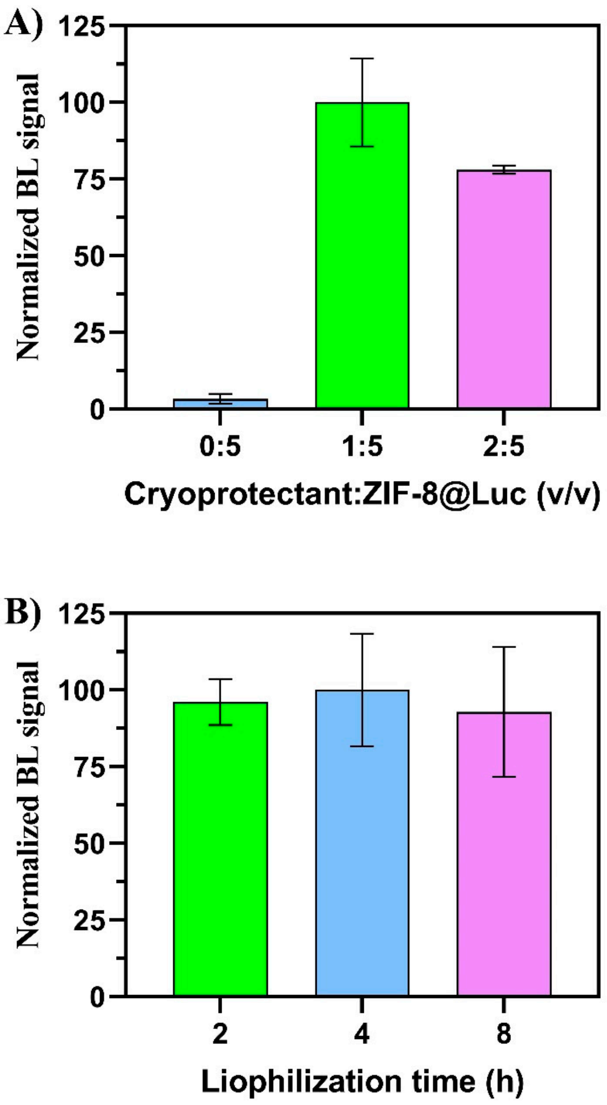

Figure S4. Method optimization - freeze drying process. A) Amount of R18 cryoprotectant used, process time 4 h. B) Time of the evaporation process, 5:1 (v/v) ratio of biocomposite:cryoprotectant. More details can be found in Materials and Methods Section.

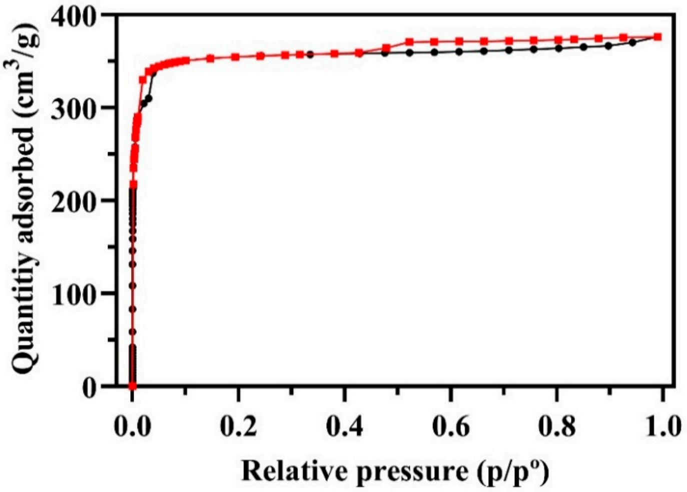

Figure S5. Nitrogen adsorption/desorption isotherms of ZIF-8. Black and red lines represent the adsorption and desorption curves, respectively. More details can be found in Materials and Methods Section.

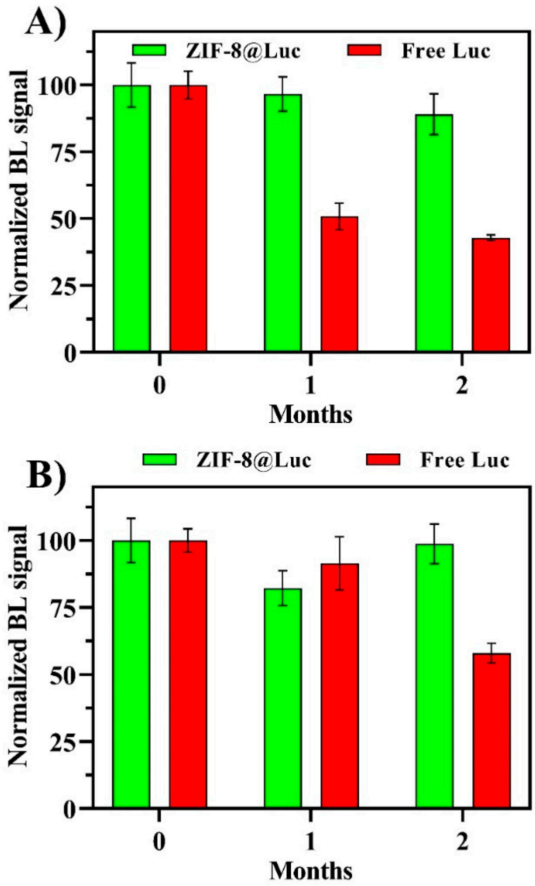

Figure S6. Long-term stability studies of the ZIF-8@Luc and free luciferase-based biosensors at A) -20 and B) 4ºC. More details can be found in Materials and Methods Section.
